# Efficiency and selectivity of CO_2_ reduction to CO on gold gas diffusion electrodes in acidic media

**DOI:** 10.1038/s41467-021-24936-6

**Published:** 2021-08-16

**Authors:** Mariana C. O. Monteiro, Matthew F. Philips, Klaas Jan P. Schouten, Marc T. M. Koper

**Affiliations:** 1 Leiden Institute of Chemistry, Leiden University, Leiden, The Netherlands; 2 Avantium, Amsterdam, The Netherlands; 3 Van’t Hoff Institute for Molecular Sciences, University of Amsterdam, Amsterdam, The Netherlands

**Keywords:** Chemical engineering, Electrocatalysis, Electrocatalysis, Nanoparticles

## Abstract

The electrochemical reduction of CO_2_ to CO is a promising technology for replacing production processes employing fossil fuels. Still, low energy efficiencies hinder the production of CO at commercial scale. CO_2_ electrolysis has mainly been performed in neutral or alkaline media, but recent fundamental work shows that high selectivities for CO can also be achieved in acidic media. Therefore, we investigate the feasibility of CO_2_ electrolysis at pH 2–4 at indrustrially relevant conditions, using 10 cm^2^ gold gas diffusion electrodes. Operating at current densities up to 200 mA cm^−2^, we obtain CO faradaic efficiencies between 80–90% in sulfate electrolyte, with a 30% improvement of the overall process energy efficiency, in comparison with neutral media. Additionally, we find that weakly hydrated cations are crucial for accomplishing high reaction rates and enabling CO_2_ electrolysis in acidic media. This study represents a step towards the application of acidic electrolyzers for CO_2_ electroreduction.

## Abstract (web summary)

Large scale CO_2_ electrolysis to produce CO has mainly been performed in neutral and alkaline media. Here, we show that it can also be realized in acidic media, with faradaic efficiencies of 80–90%, and 30% better energy efficiency than obtained in neutral media.

## Introduction

The finiteness of fossil fuel resources and the attempt to minimize the world´s CO_2_ footprint drive research towards alternative technologies for the production of fuels and chemicals. An important building block employed in large scale for the production of commodity and specialty chemicals is carbon monoxide (CO)^1^. It can be produced via the electrochemical reduction of CO_2_ (CO_2_R), and, provided the electricity comes from zero emission technologies, the carbon cycle can be closed^2^. CO is among the most economically viable products that can be obtained through the electrocatalytic reduction of CO_2_ as it has a high ratio of molecular weight per electron transferred^3–5^. Generating CO electrochemically can enable a less centralized CO production chain by allowing for on-site CO production on demand and eliminate transportation hazards and costs. Nevertheless, in order to achieve CO_2_ electrolysis to CO at a commercial scale, three main key figures of merit are desired: high current efficiency and current density, low cell potential, and no dependency of these variables on the electrode size^4^.

A known limitation of the electrocatalytic CO_2_R is the low solubility of CO_2_ in aqueous media (33 mM at 1 atm and 25 °C) and consequently the poor transport of CO_2_ to the catalyst surface^6^. This limitation can be overcome with the use of gas diffusion electrodes (GDEs), which are porous structures that contain a gas diffusion layer and a catalyst layer^7^. The electrocatalytic reaction happens at the solid-liquid-gaseous interface and the transport of reactants to the catalyst surface is highly enhanced, which allows for achieving higher reaction rates than in conventional systems. Besides the high reaction rates, in order to achieve high current efficiencies for CO, the competing hydrogen evolution (HER) reaction must be suppressed, either by engineering the catalyst/electrode^7^ or the reaction environment (electrolyte)^8,9^.

The development of CO_2_ to CO electrolyzers at relatively large scale (GDE area > 5 cm^2^) is recent, and in most work reported so far the reaction is performed in neutral to alkaline media. In general, the local concentration of various species (such as CO_2_, HCO_3_^−^, CO_3_^2−^ OH^−^, and H^+^) has shown to play a crucial role on the competition between CO_2_ reduction and hydrogen evolution, through proton^10,11^, bicarbonate^12^, or water^13^ reduction. This is relevant as, for instance, the activity of the water reduction reaction at high overpotentials may limit the window in which high current densities can be achieved while sustaining high CO current efficiencies. Industrially relevant current densities have been accomplished at GDEs with a geometrical area larger than 5 cm^2^, however, often at the expense of relatively high cell potentials^14–18^. An additional disadvantage of using neutral or alkaline media is carbonate formation, leading to significant energy and carbon losses^19^.

Because of the high activity of proton reduction at low overpotentials, the current efficiencies found for CO in acidic media are usually low, especially on electrodes with low roughness. In fact, only in a few works in the literature, CO_2_R has been investigated (at small scale) in acidic media^10,11,20,21^. Recent work from Bondue et al.^10^ has indicated that proton reduction can be fully suppressed in acidic media, as long as the rate of CO/OH^−^ formation from CO_2_R is high enough to compensate the mass transfer of protons to the electrode surface. Differential electrochemical mass spectrometry (DEMS) was used to quantify the formation of CO/H_2_ and consumption of CO_2_. The results show that with a high surface area rough gold electrode (R = 20.3), high CO_2_ pressures (high local concentration of CO_2_) allow approaching a faradaic efficiency of nearly 100% for CO in mildly acidic electrolyte. A key conclusion from that work was also that CO_2_ is reduced primarily by H_2_O, not by protons, even in acidic media, hence leading to the formation of OH^−^. However, it remains to be seen if the results from this small-scale idealized DEMS study can be translated to high-surface-area gas diffusion electrode systems. Furthermore, our recent work has shown that tailoring the electrolyte cation identity offers an opportunity to favor the water-mediated CO_2_ reduction over proton-mediated hydrogen evolution in acidic media^21^. Studies carried out in sulfate electrolytes (pH 3) using a flat gold electrode, showed that cations have no effect on the rate of proton reduction, which is only a function of the (local) proton concentration. Meanwhile, the activity for CO_2_R to CO increases as a function of the cation identity in the order Li^+^ < Na^+^ < K^+^ < Cs^+^. In addition, performing CO_2_R in acidic media presents several advantages to a large scale process in terms, for example, of the electrolyte conductivity, HCO_3_^−^ crossover, anode reaction kinetics (in case of oxygen evolution) and electrolyzer design.

In this work we have assessed the feasibility of acidic CO_2_ electrolysis with practical electrode geometries. Using a 10 cm^2^ gold gas diffusion electrode, we performed CO_2_R in sulfate electrolytes at different current densities (10–200 mA cm^−2^). Our results show that high CO selectivity (~90%) can be achieved at 100–200 mA cm^−2^ in mildly acidic media, as long as weakly hydrated cations are present in the electrolyte (Cs^+^, K^+^). Through a direct comparison with neutral media (1 M KHCO_3_) we find that in 1 M Cs_2_SO_4_ electrolyte, CO_2_R can be carried out at considerably lower cell potentials, leading to a reduction of up to 30% in process energy costs. Our work represents an important step towards acidic CO_2_ electrolysis at industrial conditions, with improved energy efficiency.

## Results

**Fig. 1:**
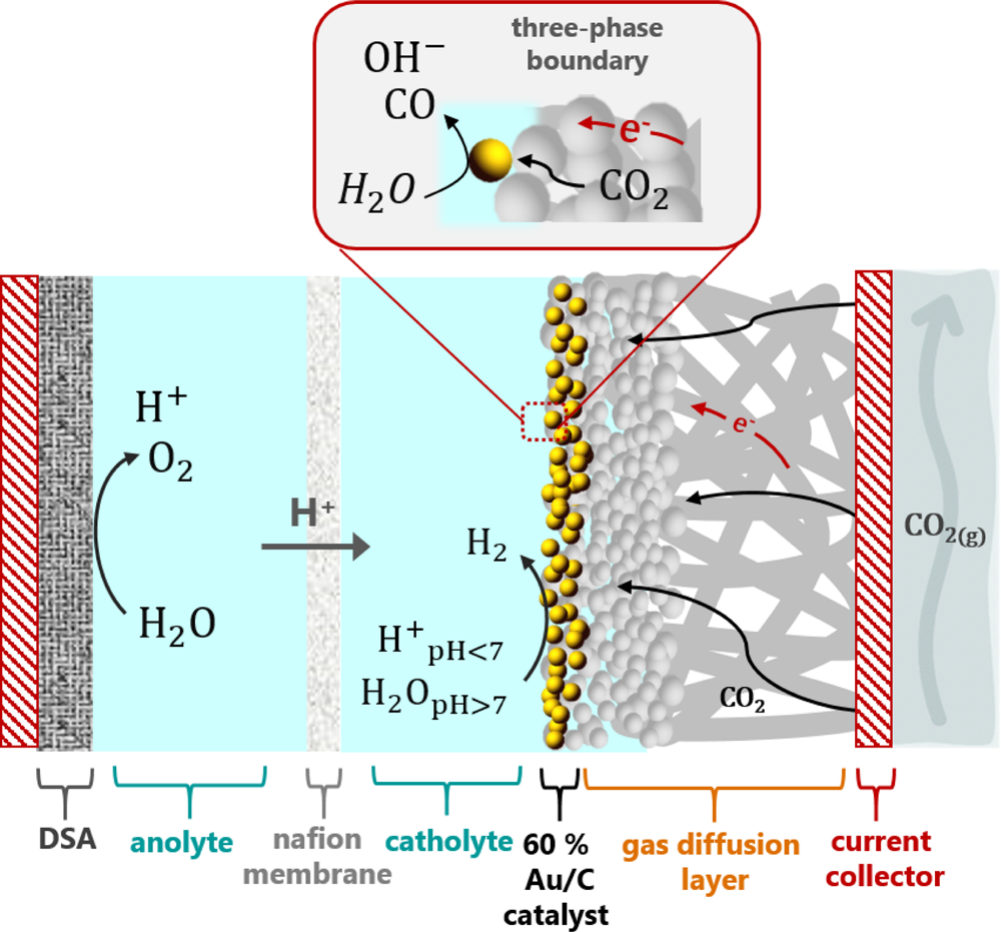
CO_2_ electrolysis. Schematic representation of the gold gas diffusion electrode system studied in this work.

CO_2_ reduction (CO_2_R) was carried out on gold gas diffusion electrodes, whose schematic structure is depicted in Fig. 1. At the gold-based cathode, CO_2_ is fed through the back of the electrode and reduced to CO (CO_2_ + H_2_O + 2e^–^ → CO + 2OH^−^) at a three phase boundary between the catalyst, the catholyte and CO_2(g)_. At the DSA^®^ anode, water is oxidized to O_2_ (H_2_O → ½O_2_ + 2H^+^ + 2e^–^). Depending on the reaction conditions, not all the CO_2_ supplied to the cathode is converted to CO. Hydrogen (H_2_) can competitively form either through proton reduction or water reduction, depending on the acidity of the local reaction environment. The local pH near the cathode surface can change significantly as a function of the current density and reaction selectivity. However, the OH^−^ produced during CO_2_ reduction can be neutralized by protons (if the catholyte is acidic) or by the reactant itself, succesively forming bicarbonate and carbonate (CO_2_ + OH^−^ → HCO_3_^−^; HCO_3_^−^ + OH^−^ → CO_3_^2−^ + H_2_O). Because of these homogeneous reactions, it is important to supply an excess of CO_2_ in order to achieve high current densities and CO faradaic efficiencies. For all the measurements described in this work, the flow of CO_2_ (50 mL min^−1^) and electrolyte (30 mL min^−1^) were kept constant, and the anolyte was always 0.5 M H_2_SO_4_. The setup did not allow the introduction of a reference electrode, but all system parameters were kept constant in order to allow a comparison of the effect of electrolyte pH on the cathodic reaction. That is: the only parameters varied were on the cathode side, where different electrolytes were investigated and two different loadings of gold nanoparticles were applied to 10 cm^2^ GDEs, namely 2 and 1 mg cm^−2^.

**Fig. 2:**
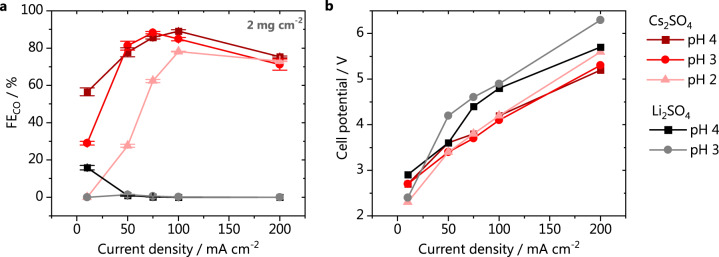
Effect of pH and cation identity on CO_2_ electrolyis. **a** Faradaic efficiency for CO and (**b**) cell potential. Electrolysis performed in either 1 M Cs_2_SO_4_ (red) or 1 M Li_2_SO_4_ (gray scale), catalyst loading 2 mg cm^−2^.

We have first investigated the feasibility of carrying out CO_2_ electrolysis on GDEs in acidic media, by performing bulk electrolysis in 1 M Cs_2_SO_4_ solutions of pH 2, 3 and 4. The faradaic efficiencies (FE) for CO as a function of the total applied current density can be seen in Fig. 2a. The only products formed were CO and H_2_, evidenced by the CO and H_2_ FEs adding up to 100% (Supplementary Fig. 1). Even though formate has been reported as a possible product from CO_2_ reduction on gold electrodes^22^, it was not detected under our working conditions (if it was formed it was below our detection limit). At low current densities, we see that the selectivity for CO is lower at the more acidic electrolyte than at pH 4. However, at 200 mA cm^−2^ >70% FE for CO is obtained, regardless of the bulk pH. A FE of 90% is achieved at pH 4, at 100 mA cm^−2^. In terms of selectivity, these results not only show that large scale CO_2_R to CO can be accomplished in acidic media, but are also in agreement with the model recently proposed by Bondue et al. based on small-scale DEMS measurements^10^. However, in our case, at low current densities, and low pH, due to the lower mass transport limitation in the GDE configuration, the activity for CO (and corresponding production of OH^−^) is not high enough to neutralize the protons diffusing towards the catalyst surface, favouring hydrogen production. On the other hand, at current densities above 50 mA cm^−2^, especially for bulk pH 3 or 4, the local acidic environment can be rapidly neutralized^23,24^, enabling FEs for CO above 80%. We see that when starting from bulk pH 2, higher current densities are required to achieve this suppression of proton reduction. The slight decrease in selectivity for CO going from 150 to 200 mA cm^−2^ is likely due to the strong alkalinity developing around the catalyst (depletion of CO_2_ due to the formation of carbonate) and the onset potential of the water reduction reaction. When operating electrolyzers at high (>100 mA cm^−2^) current densities, low energy costs are crucial to make the process feasible. The cell potentials obtained at pH 2–4 are shown in Fig. 2b, and no significant differences are observed as a function of pH at intermediate current densities, where the selectivities for CO are high. However, at 10 mA cm^−2^, a lower cell potential is found at pH 2, as mainly proton reduction is taking place. The inverse happens at 200 mA cm^−2^, likely due to the lower activity of water reduction. We point out that the measurements at different current densities were performed sequentially, and the GDEs were stable throughout the experiments, i.e., no problems with flooding were encountered.

The cation identity has also been shown to play an important role on the selectivity and activity of CO_2_ reduction. In neutral and alkaline media, Bhargava et al.^25^ have recently reported FEs for CO on silver GDEs increasing from 40 up to nearly 100% depending on the cation used, in the order Na^+^ < K^+^ < Cs^+^ at 50 mA cm^−2^. In acidic media, we have recently shown that the cation identity does not affect the activity for proton reduction, while weakly hydrated cations lead to a drastic increase in the activity for CO^21^. We showed that the cation acts as a necessary promotor for the reaction and that the cation trend for CO formation is related to the higher ability that weakly hydrated cations, such as Cs^+^, have to coordinate with the adsorbed CO_2_^−^ intermediate, and their high concentration near the electrode surface. In contrast, cations having a strong hydration shell such as Li^+^, do not strongly coordinate with the oxygen of the adsorbed CO_2_, and accumulate less near the surface, leading to their inferior promoting effect on the CO production^21^. Therefore, we have also investigated to which extent the electrolyte cation identity plays a role when carrying out CO_2_R in acidic media in the GDE geometry. The results obtained in 1 M Li_2_SO_4_ at pH 3 and 4 are compared to Cs_2_SO_4_ in Fig. 2a and the total FEs for CO and H_2_ are available in Supplementary Fig. 2. Surprisingly, we observe that in Li_2_SO_4_ nearly no CO is produced at all applied current densities, regardless of the bulk pH. The low activity for CO_2_R to CO in Li_2_SO_4_ does not allow for reaching high enough CO_2_R currents at low overpotentials and to hinder the access of protons to the catalytic active sites, yielding nearly 100% FEs for H_2_ at these current densities. This is confirmed by the higher cell potentials that are observed in nearly the whole current range investigated (Fig. 2b). These findings highlight that differently from neutral and alkaline media, the choice of a cation that favors the production of CO is crucial to enable CO_2_ electrolysis in acidic media.

**Fig. 3:**
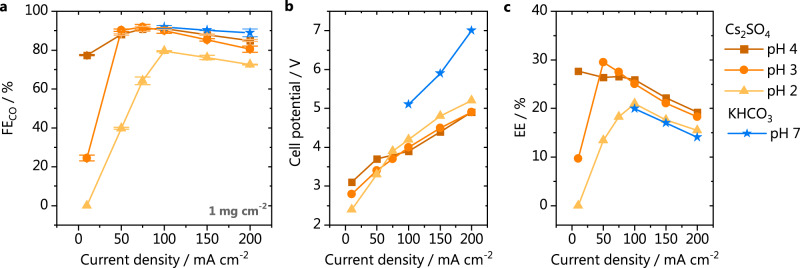
Comparison between acidic and neutral media. **a** Faradaic efficiency for CO; (**b**) cell potential and (**c**) energy efficiency for CO_2_ electrolysis performed in either 1 M Cs_2_SO_4_ (orange) or 1 M KHCO_3_ (blue stars), catalyst loading 1 mg cm^−2^.

**Fig. 4:**
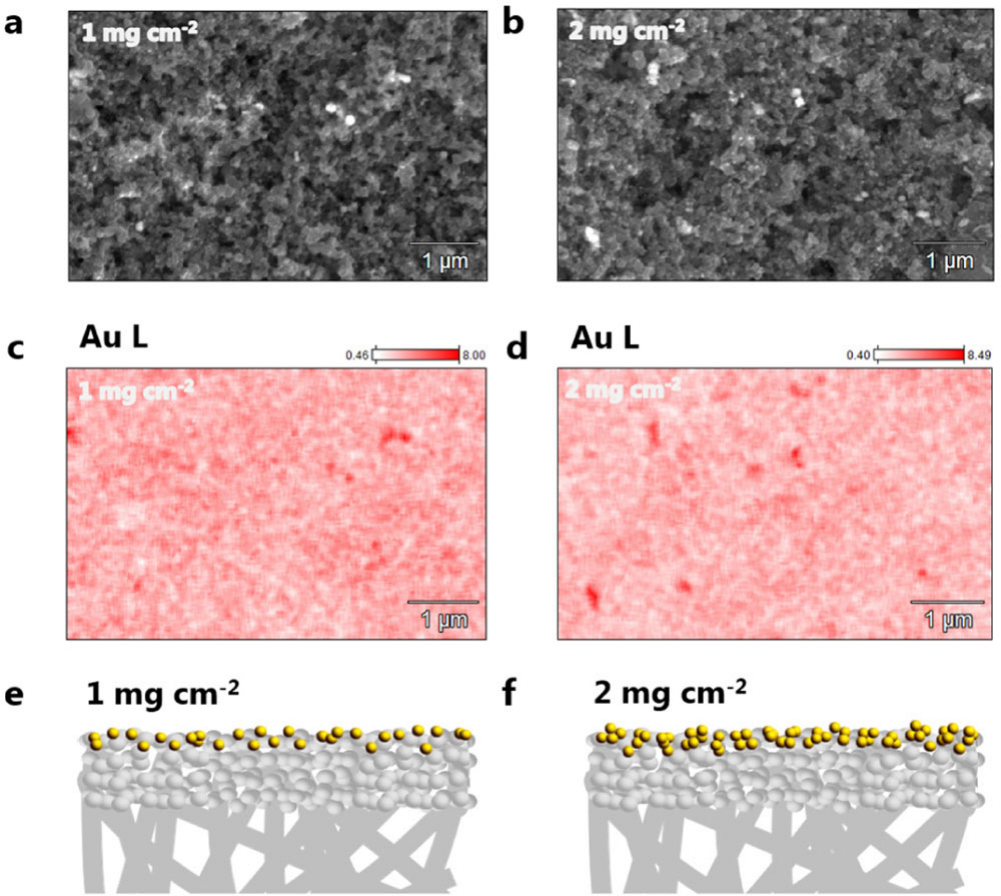
Characterization of the GDEs with different catalyst loadings. **a**, **b** SEM micrographs, (**c**, **d**) EDX elemental map and (**e**, **f**) schematic representation of the gold GDEs with 1 mg cm^−2^ and 2 mg cm^−2^ loading, respectively.

We have also prepared a GDE with half the loading of gold nanoparticles than the one used in the experiments shown in Fig. 2. CO_2_ electrolysis was carried out at the gold GDE with 1 mg cm^−2^ loading in the same conditions as before (1 M Cs_2_SO_4_, pH 2–4) and the FEs for CO and cell voltages are displayed in Fig. 3. The total faradaic efficiencies for CO and H_2_ are shown in Supplementary Fig. 3. Figure 3a shows the same FE trend observed for the 2 mg cm^−2^ GDE as a function of pH. However, the overall values are slightly higher for the lower loading GDE, independent of the bulk pH (see a direct comparison in Supplementary Fig. 4). There are several structural factors of the GDE that could lead to the observed differences. The dispersion of the nanoparticles, for instance, could affect how accessible the catalytic sites are for the build up of the three-phase reaction boundary. Significant differences in thickness of the GDE could also alter the potential drop across the layer and consequently the activity/selectivity. In the literature, there is no consensus about the exact effect that changing the catalyst loading has on the reaction. The work of Bhargava et al.^25^ with Ag-based 1 cm^2^ GDEs suggests that no significant differences are found in terms of CO faradaic efficiency for loadings above 1 mg cm^−2^. In the work of Duarte et al.^17^, on the other hand, using 10 cm^2^ Ag-based GDEs, a higher activity for CO was found in potentiostatic measurements when increasing the catalyst loading from 0.5 to 2 mg cm^−2^. Nevertheless, no significant changes in selectivity were observed. The differences in GDE dimensions and production procedures, in addition to the complex morphologies of these electrodes, currently do not allow drawing a general rule in terms of the effect of the catalyst loading. In view of that, we have analyzed our specific case, by characterizing the morphology and composition of the GDEs used in this work using Scanning Electron Microscopy (SEM) and Energy Dispersive X-Ray Spectroscopy (EDX). The SEM micrographs for the GDEs with 1 and 2 mg cm^−2^ loading can be seen in Fig. 4a and Fig. 4b, respectively. There are no clear differences between the overall morphology of the two GDEs, which can be also seen in the additonal micrographs from Supplementary Fig. 5. However, looking at the dispersion of the gold nanoparticles, it becomes clear that more particle agglomerates are present at the surface of the GDE with the higher catalyst loading. This is further confirmed by elemental analysis (Fig. 4c, d), implying that at the GDE with a lower loading, the nanoparticles are better dispersed on the PTFE matrix. Even though more nanoparticles are present in the GDE with higher loading, these particles are likely less accessible for the reactants, hindering the formation of the three-phase reaction boundary. A schematic representation of these differences is depicted in Fig. 4e, f. It is important to point out that although only one micrograph is shown here, several areas of the GDEs were imaged in order to draw these conclusions. The images shown here are representative of the trend found throughout the whole surface. The complete EDX elemental analysis of the GDEs is shown in Supplementary Figs. 6 and 7. The two GDEs have nearly the same composition, in which carbon and fluorine are present in large amounts, due to the PTFE-based gas diffusion layer. In addition, as the analysis was performed after the catalysts were used for CO_2_ electrolysis (for obtaining a more realistic picture of the GDE during/after operation) cesium and potassium hydroxide deposits are also found homogeneouly distributed in the GDE structure, due to the extreme alkalinity developed at high current densities.

The performance of the 1 mg cm^−2^ GDE in acidic media was compared with its performance in neutral media, by carrying out the electrolysis in 1 M KHCO_3_, a commonly used electrolyte for CO_2_R. The total FEs for CO and H_2_ in KHCO_3_ are shown in Supplementary Fig. 8. The FEs for CO are compared in Fig. 3a, where we see that the selectivity in sulfate, especially at pH 3 and 4, is nearly the same as in KHCO_3_ for current densities between 100–200 mA cm^−2^. However, by comparing the respective cell potentials (Fig. 3b), we find remarkably lower values in acidic media. This is likely a consequence of the much higher conductivity of the 1 M Cs_2_SO_4_ (234.4 10^−4^ S m^2^ mol^−1^) electrolyte in comparison to 1 M KHCO_3_ (117.9 10^−4^ S m^2^ mol^−1^), which decreases the ohmic losses. Considering that the electricity is the main operating cost of CO_2_ electrolysis^5^, it is crucial that the energy efficiency is optimized in order to make the technology industrially viable. It can be seen in Fig. 3c, that operating the cathode in acidic media, in sulfate electrolyte, leads to a considerable improvement of the process energy efficiency compared to KHCO_3_ (see Supplementary Equation 1). A similar comparison in terms of the energy consumption per ton of CO produced can be made (Supplementary Fig. 9) and shows that running CO_2_ electrolysis in Cs_2_SO_4_ at pH 4 instead of KHCO_3_ (both 1 M) leads to an energy saving of 24, 25 and 30% at 100, 150, and 200 mA cm^−2^, respectively. Considering, for example, an electricity price of 0.03 dollars per kWh^5^, running the reaction in Cs_2_SO_4_ at pH 4 instead of KHCO_3_ (at 200 mA cm^−2^) could lead to saving 1343 dollars per ton of CO produced. The process energetics in sulfate could of course be improved even further, by increasing the ionic strength of the electrolyte in order to reduce ohmic losses, and by refining the cell design.

**Fig. 5:**
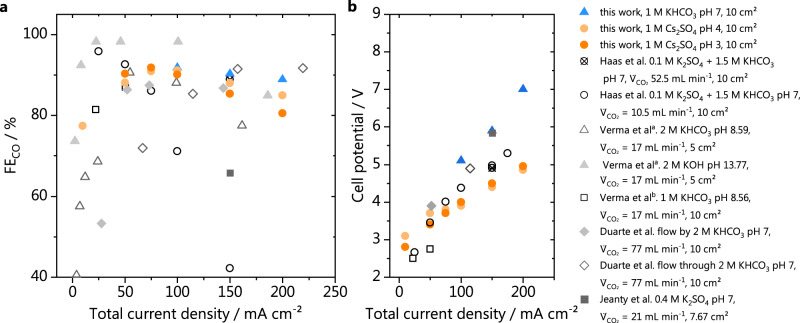
Comparison of our results in acidic media with literature values. **a** Faradaic efficiencies and (**b**) cell potentials reported in different work from literature, in which CO_2_ electrolysis was performed in neutral to alkaline media, using GDEs equal or larger than 5 cm^2^. Results from this work shown in orange circles (acidic media) and blue triangles (neutral media). Data points extracted from the work of Haas et al.^15^, Verma et al.^a^ ^13^, Verma at al.^b^ ^14^, Duarte et al.^16^ and Jeanty at al.^17^ shown in gray.

We have also compared the results of our work, performed in acidic media, with recently reported literature values, for CO_2_R to CO in neutral/alkaline media performed in gas diffusion electrodes having a geometrical area ≥ 5 cm^2^. It must be noted that although CO_2_R is intensively studied, mainly potentiostatic experiments are carried out, which are not representative of an electrocatalytic industrial process. Often, only partial current densities for CO are reported, and no information regarding the cell potential is given. This creates a gap in terms of determining the economical feasibility of the process, and for comparing different reaction conditions and catalysts. Therefore, we have only compared here results obtained in (fairly) similar systems to the one of this work. The main difference is that, in all works reported so far, CO_2_R to CO has been carried out in catholytes of pH 7 or higher. We have only compared measurements performed at similar scale (5–10 cm^2^ GDEs) and in electrolytes with moderate to high ionic strength (0.5–2 M). The collection of data points is shown in Fig. 5, for the CO faradaic efficiencies and cell potentials as a function of the total current density. We see in Fig. 5a that in terms of selectivity, the performance of our GDEs at acidic pH is quite comparable to results obtained in neutral and alkaline media, especially at the higher current densities. On the other hand, in Fig. 5b it can be seen that the cell potentials we obtained in acidic media are considerably lower than the literature values, supporting what we have also concluded based on our own comparison with KHCO_3_.

## Discussion

Based on our results in acidic media, the system design rules for CO_2_ electrolyzers, recently presented by Bhagarva et al.^25^, should be reconsidered. In their work, pH 14 is pointed out as nearly the sole condition that can yield optimum CO_2_R to CO performance. However, their comparison is based only on experiments carried out in 1 cm^2^ GDEs in electrolytes with pH higher than 6. Our work shows that operating in acidic media, specifically in sulfate electrolyte, can considerably decrease the energy costs without harming other important figures of merit such as current density and FE. Table 1 gives an overview of the molar conductivities of different anions and cations that have been used/considered to carry out CO_2_R. It can be seen that an acidic electrolyte (of a given cation) will always have higher conductivity than an alkaline one. In addition, the less bicarbonate formed during the course of the reaction, the better it is in terms of carbon and efficiency losses. In acidic media, the protons can to some extent neutralize the OH^−^ produced upon reduction of CO_2_ while in alkaline media these species will be neutralized by CO_2_ itself, lowering the reactant´s concentration at the reaction interface. Although pH 14 or larger has been proposed as the optimum condition to carry out CO_2_ electrolysis^25^, it has been recently emphasized that a series of difficulties can arise from operating in hydroxide-based electrolytes at large scale. The work from Nesbit and Smith^26^ shows that at high current densities (>1 A cm^−2^) there is a local increase in the electrolyte concentration, which consequently increases its viscosity, slows down diffusion, lowers the electrolyte conductivity (e.g., for KOH concentration >5 M) and CO_2_ solubility, increases the rate of HCO_3_^−^ production, increases the solute activity and lowers the activity of water. According to the authors, these factors slow down the rate of CO_2_R and shift the reaction equilibrium potentials. These contributions would be much less significant if operating in an acidic electrolyte. In addition, it is important to point out a few considerations that must be made in terms of the choice of anion for carrying out CO_2_ electrolysis in acidic media. As shown in Table 1, sulfate anions not only have the highest conductivity (after OH^−^) but also allow for working in highly concentrated electrolyte. A potassium sulfate solution, for example, can be prepared in concentrations up to 6.8 M. This is not the case for perchlorate, for example, which despite its use in many fundamental electrochemical studies, is poorly soluble and could never be employed in a large scale process. Analyzing Table 1 further, halide anions would be an option considering their high solubility, however, it has been shown that specific adsorption is detrimental for the activity for CO_2_ reduction. Finally, although hydrogenphosphate has relatively good conductivity and solubility, it has been proposed to act as a proton donor for hydrogen evolution^27^.

**Table 1:** Electrolyte compositions for CO_2_ electrolysis.

Conductivity				
cation	Λ_+_ 10^−4^·S·m^2^·mol^−1^		anion	Λ−10^−^^4^·S·m^2^·mol^−1^
H^+^	349.6		OH^−^	198
Rb^+^	77.8		SO_4_^2−^	160
Cs^+^	77.2		CO_3_^2−^	138.6
K^+^	73.4		HPO_4_^2−^	114
Na^+^	50.1		Br^−^	78.1
Li^+^	38.7		I^−^	76.8
			Cl^−^	76.3
			NO_3_^−^	71.4
			ClO_4_^−^	67.3
			F^−^	55.4
			HCO_3_^−^	44.5
			H_2_PO_4_^−^	36
**Solubility**				
**salt**		molal*	**salt**	molal*
KOH		21.6	K_2_HPO_4_	8.6
K_2_SO_4_		6.9	KBr	45.0
K_2_CO_3_		8.0	KNO_3_	31.3
KHCO_3_		2.2	KClO_4_	0.1

Equivalent ionic conductivity^29^ (Λ°) of commonly used electrolyte ions, and feasible molality based on the solubility^30^ of potassium salts containing different anions.

*Molality (mol kg^−1^) calculated assuming the maximum concentrations feasible according to the solubility values at 20 °C^30^.

In summary, here we have shown that, while most of the efforts in the CO_2_R field have been on developing better cathode catalysts, the electrolyte design, specifically operating in acidic media, can considerably lower the cell potential and consequently improve the energy efficiency, without compromising the FE for CO. Based on the recent observation in idealized small-scale DEMS measurements that under suitable conditions proton reduction can be suppressed during CO_2_R, our results show that FEs between 80–90% for CO can be achieved at a gold gas-diffusion electrode at current densities up to 200 mA cm^−2^, demonstrating the feasibility of running CO_2_ electrolysis in acidic media. Under these industrially relevant conditions, the high rate of CO_2_ reduction (i.e., high current density) leads to the production of sufficient OH^−^ to neutralize the protons before they reach the catalyst layer. This hinders proton reduction and enables a high selectivity for CO even at bulk pH 2, at 200 mA cm^−2^. Furthermore, we observe that employing weakly hydrated cations in the electrolyte, such as Cs^+^ and K^+^, is crucial for assuring a high CO_2_ reduction activity in acidic media. Considering operation at industrially relevant scales, there must be a tradeoff between performance and cost, which means K^+^ sulfate salts are likely more suitable. Finally, by comparing results in acidic and neutral media, we show that considerably lower cell potentials can be achieved at low pH in sulfate electrolyte, which result in energy savings up to 30% operating at 200 mA cm^−2^. Further research is of course necessary in order to lower energy costs even more, achieve higher current densities and fully optimize the process. Our work, however, lays down a new path towards the development of acidic CO_2_ electrolyzers.

## Methods

### Materials and chemicals

The following chemicals were used to prepare the electrolytes for bulk CO_2_ electrolysis: Cs_2_SO_4_ (Sigma Aldrich, 98%), Li_2_SO_4_ (Sigma Aldrich, 98%), KHCO_3_ (Acros Organics, 99.5%), H_2_SO_4_ (Acros Organics, for analysis ACS, 95% solution in water).

### Gas diffusion electrodes fabrication

The synthesis method for the gas diffusion layer (GDL) was adjusted from a patented method^28^. First, 15 g of acetylene black (Soltex, 75%-03) was mixed with 8.91 mL PTFE (FuelCellStore, Teflon™ PTFE DISP 30) and 60 mL of a 1:1 volume water/isopropyl alcohol (IPA). After mixing, a dough was obtained and rolled with a marble rolling pin before using a cross rolling technique to obtain the desired thickness. The PTFE dispersion was diluted by 50% with 1:1 volume water/IPA, applied to the back of the dough, and graphitized carbon was placed on top. A Carver heated press (Model number 4533) was used to press the structure at 140 °C and 10.25 Ton for 32.5 min. Then the temperature was raised to 308 °C at the same pressure and held for 32.5 min. Finally the temperature was raised to 317.5 °C at 13 ton and held for 32.5 min before removing the structure from the press. GDLs of 17.16 cm^2^ were cut from the resulting structure. Commercial gold nanoparticles were used as catalyst (60% gold supported on vulcan XC-72 (carbon), FuelCellStore). The catalyst ink was prepared by suspending the particles in ethanol and adding 250 μL of a Nafion^©^ solution under constant stirring. The inks were additionally sonicated for 30 min. The obtained ink was airbrushed on a 17 cm^2^ GDL and let dry. The catalysts metal loading was calculated by weighing the electrodes before and after the spraying.

### Gas diffusion electrodes characterization

The gold GDEs topography and composition were characterized by Scanning Electron Microscopy (SEM) in an Apreo SEM (ThermoFisher Scientific). Micrographs were obtained using an acceleration voltage of 10 kV and an electron beam current of 0.8 nA. Energy Dispersive X-Ray Spectroscopy (EDX) was used for elemental analysis (Oxford Instruments X-MaxN 150 Silicon Drift detector). EDX data processing was done with the Pathfinder™ X-ray Microanalysis software v1.3. The data is displayed in atomic percentage for easier visualization, however, the quantification was performed in automatic mode, without providing external standards.

### Electrolysis measurements

The gas diffusion electrodes were mounted in a commercial two-compartment 10 cm^2^ GDE flow cell (ElectroCell, Micro Flow Cell) for the bulk electrolysis. The anolyte and the catholyte were separated by a reinforced Nafion^TM^ membrane N324. For all measurements the anolyte was a 0.5 M H_2_SO_4_ solution and the anode a dimensionally stable anode (DSA^®^, ElectroCell). The electrolyte flow rate was 30 mL min^−1^, achieved with a peristaltic pump. A CO_2_ flow of 50 mL min^−1^ was employed for all the measurements. Either 1 M Cs_2_SO_4_, Li_2_SO_4_, or KHCO_3_ were used as catholyte. The pH of the solutions was adjusted with H_2_SO_4_ when necessary using a pH meter. Galvanostatic bulk electrolysis measurements were controlled using a power supply and each different current density was applied for one hour. The product analysis was performed using a gas chromatograph (Varian 4900 micro GC) equipped with four modules: COx module, MS5 (mol. sieve) module, PPQ (poraplotQ) module and 52 C WAX module. Gaseous samples were taken from the headspace every 3 min. The current efficiencies shown throughout this work represent the average values obtained during 1 h of electrolysis, with the corresponding standard deviation.

## Supplementary information

Supplementary Information

Peer Review File

## Data Availability

Experimental datasets are available from the corresponding author on reasonable request.
