# Supplementary material for: Efficiency and selectivity of CO2 reduction to CO on gold gas diffusion electrodes in acidic media
Source: Nat Commun. 2021 Aug 16;12:4943. doi: 10.1038/s41467-021-24936-6 (PMC8368099; doi:10.1038/s41467-021-24936-6)
Supplement: Supplementary file 1 — Supplementary Information [file 41467_2021_24936_MOESM1_ESM.pdf]

Efficiency and selectivity CO_2 reduction to CO on gold gas diffusion electrodes in acidic media

Mariana C. O. Monteiro[a], Matthew F. Philips[a,b], Klaas Jan P. Schouten[b,c,*], Marc T. M. Koper[a,*]

[a] Leiden Institute of Chemistry, Leiden University, P.O. Box 9502, 2300 RA, Leiden, the Netherlands

[b] Avantium, Science Park 408, 1098 XH Amsterdam, The Netherlands

[c] Van't Hoff Institute for Molecular Sciences, University of Amsterdam, P.O. Box 94157, 1090 GD Amsterdam, The Netherlands

Supplementary Information

1. Effect of pH

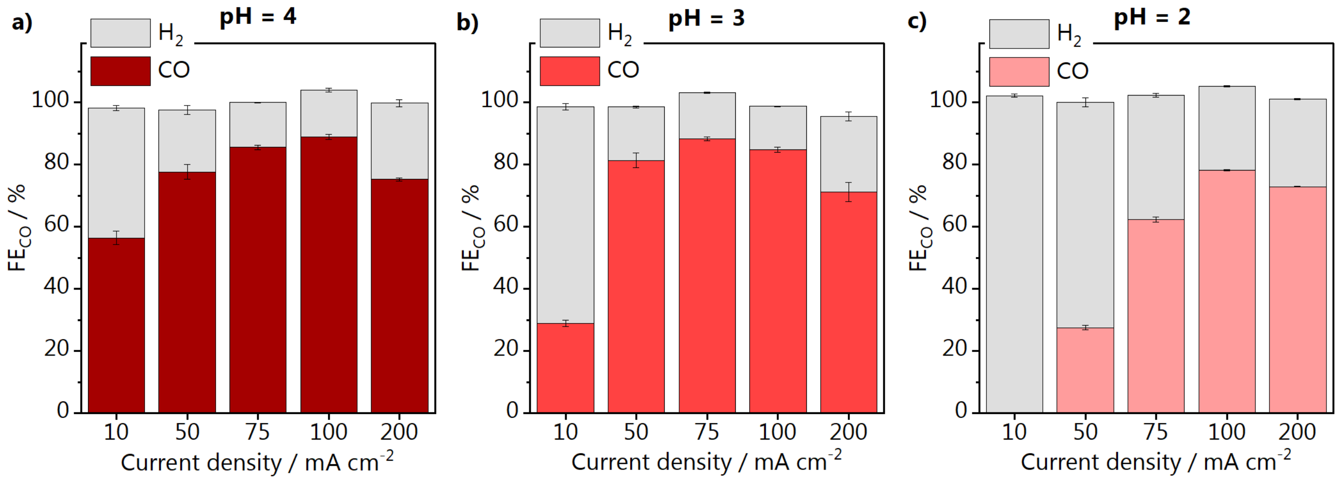

Supplementary Figure 1. Faradaic efficiencies for CO and H_2 obtained during one hour electrolysis at each current density displayed. The catholyte was 1 M Cs_2SO_4, **a)** pH 4, **b)** pH 3 and **c)** pH 2. Catalyst loading 2 mg cm^{-2}. Error bars are calculated based on three individual measurements.

2. Cation identity

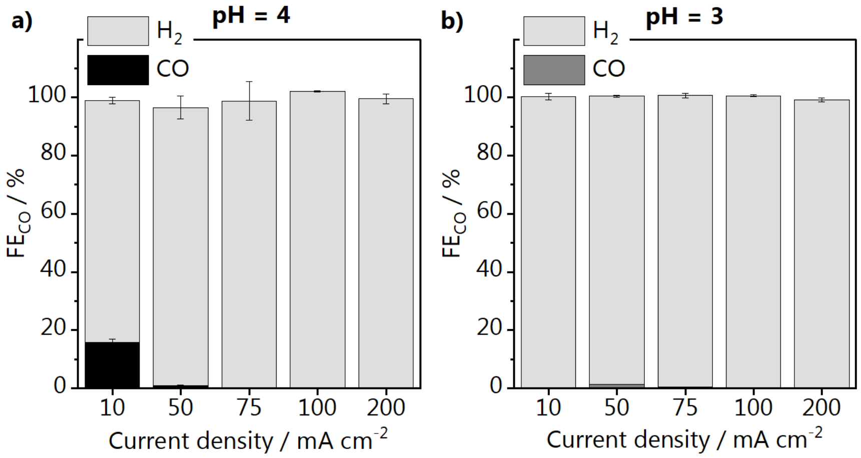

Supplementary Figure 2. Faradaic efficiencies for CO and H_2 obtained during one hour electrolysis at each current density displayed. The catholyte was 1 M Li_2SO_4, **a)** pH 4 and **b)** pH 3. Catalyst loading 2 mg cm^{-2}. Error bars are calculated based on three individual measurements.

3. Effect of the catalyst loading

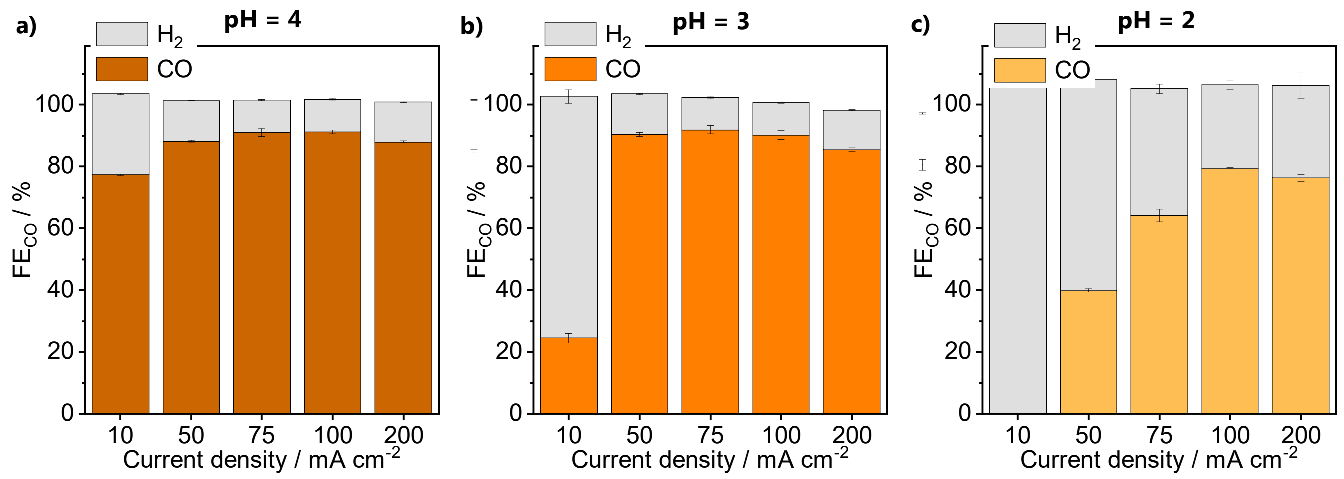

Supplementary Figure 3. Faradaic efficiencies for CO and H_2 obtained during one hour electrolysis at each current density displayed. The catholyte was 1 M Cs_2SO_4; **a)** pH 4, **b)** pH 3 and **c)** pH 2. Catalyst loading 1 mg cm^{-2}. Error bars are calculated based on three individual measurements.

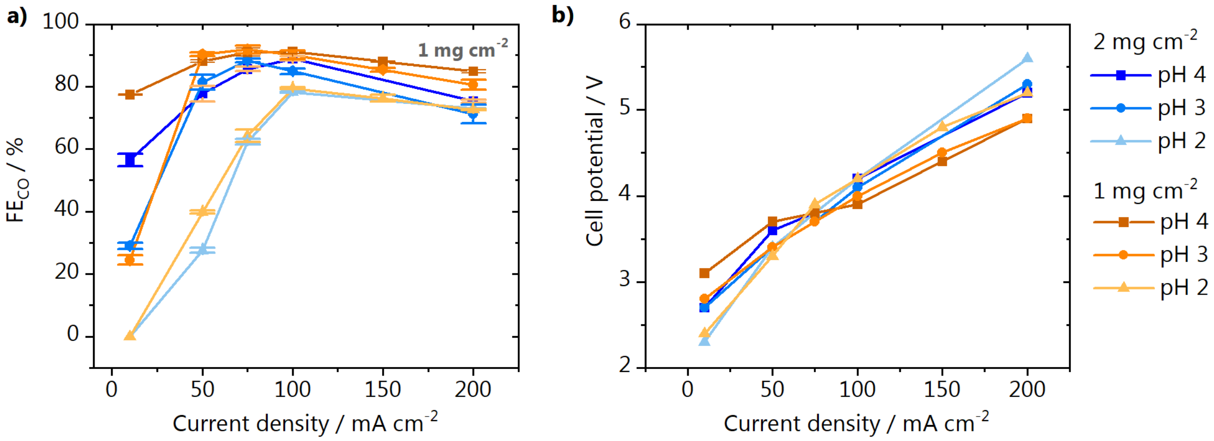

Supplementary Figure 4. Electrolysis performed in 1 M Cs_2SO_4, catalyst loading 1 mg cm^{-2} or 2 mg cm^{-2}, **a)** faradaic efficiency for CO and **b)** cell potential. The data points displayed are an average obtained during 1 hour electrolysis. Error bars are calculated based on three individual measurements.

4. SEM micrographs and EDX elemental analysis of the GDEs with different loadings.

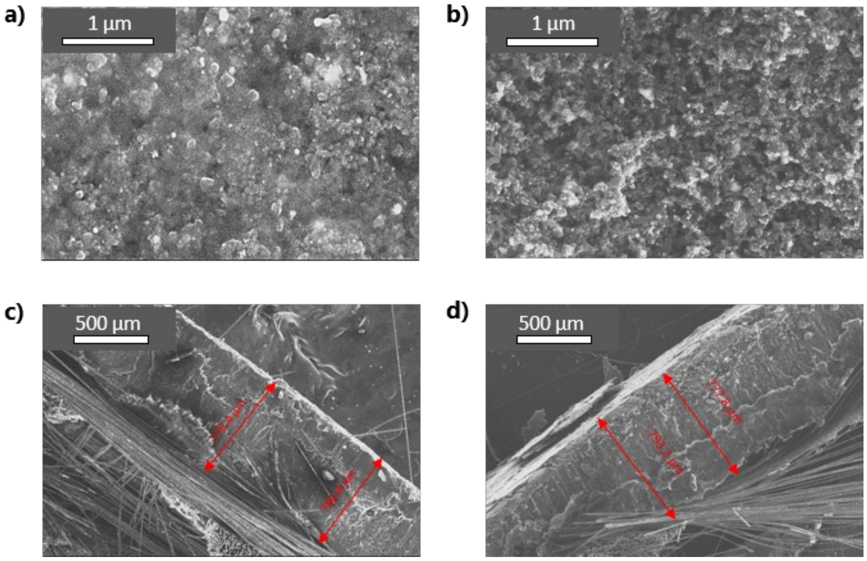

Supplementary Figure 5. SEM micrograph of the gold GDEs. Top view of the **a)** 1 mg cm^{-2} and **b)** 2 mg cm^{-2} catalyst, and cross-section of the **c)** 1 mg cm^{-2} and **d)** 2 mg cm^{-2} catalyst.

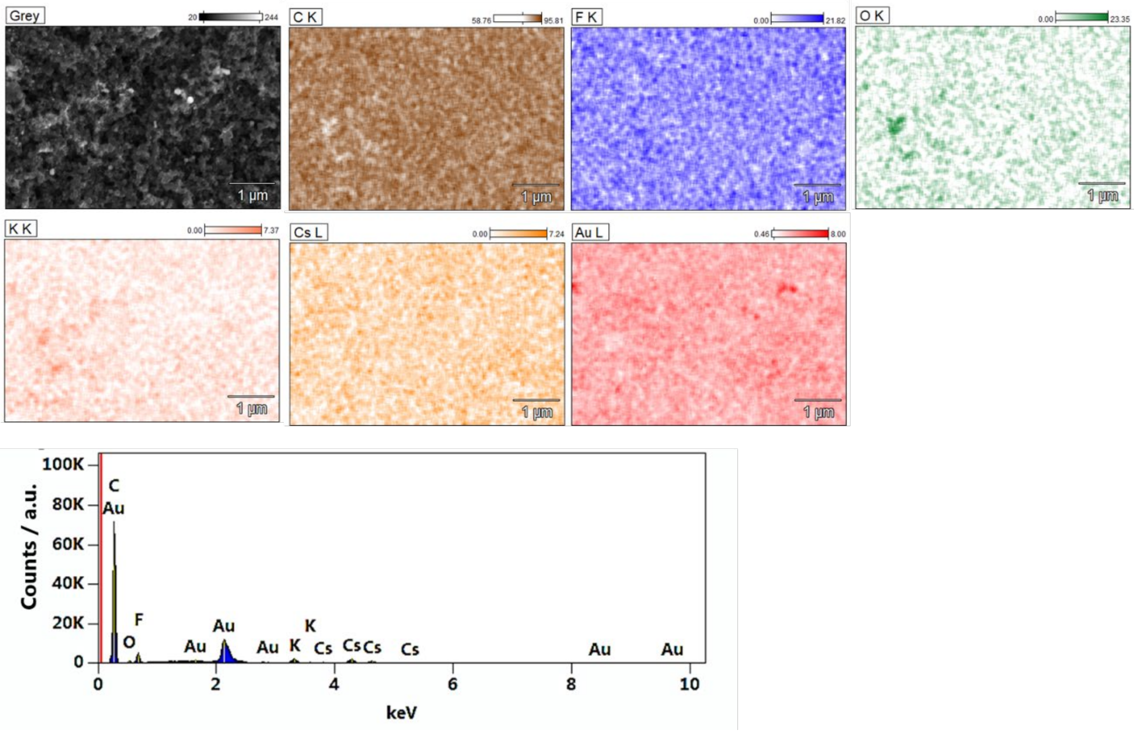

Supplementary Figure 6. SEM micrograph, EDX elemental map and spectrum of the gold GDE with 1 mg cm^{-2} loading.

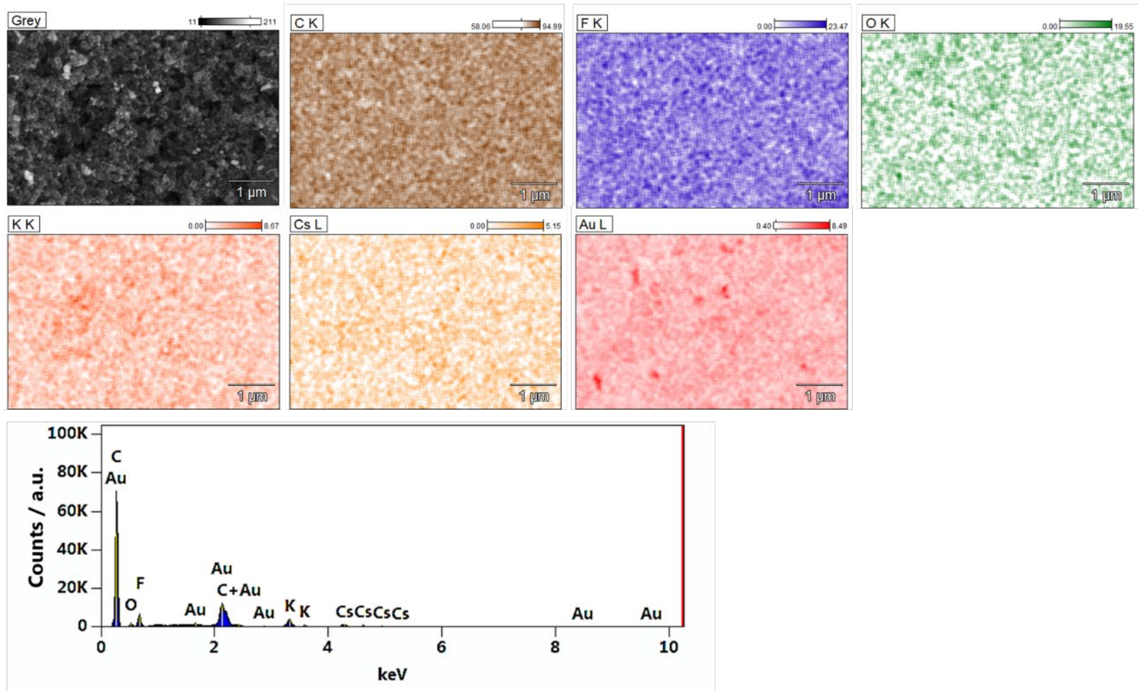

Supplementary Figure 7. SEM micrograph, EDX elemental map and spectrum of the gold GDE with 2 mg cm^{-2} loading.

5. Comparison with bicarbonate

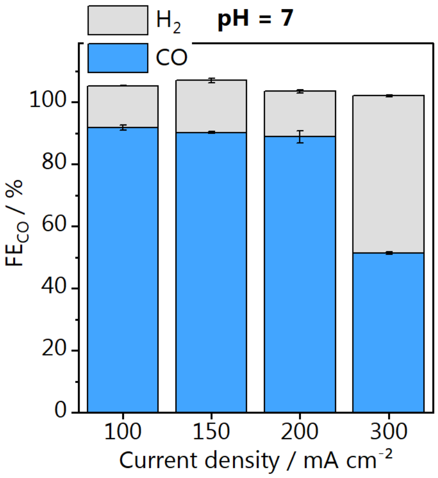

Supplementary Figure 8. Faradaic efficiencies for CO and H_2 obtained during one hour electrolysis at each current density displayed. The catholyte was 1 M $KHCO_3$ (pH 7) and the catalyst loading 1 mg cm^{-2}. Error bars are calculated based on three individual measurements.

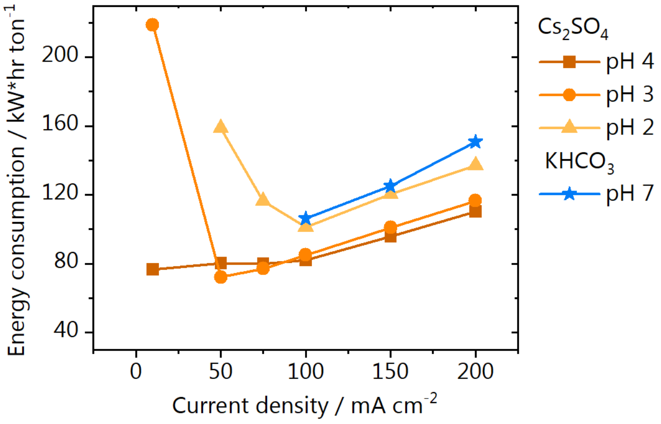

Supplementary Figure 9. Energy consumption per ton of CO produced, based on the faradaic efficiencies and cell potentials obtained for electrolysis in 1 M Cs_2SO_4 or 1 M $KHCO_3$ at different current densities (catalyst loading 2 mg cm^{-2}).

Energy efficiency calculation:

$$\varepsilon_{energy} = \frac{\left(E^0_{CO_2} + E^0_{O_2}\right) * FE_{CO}}{E_{Cell}}$$ Supplementary Equation 1

where E^0 are the standard potentials of the cathode and anode reactions, *FE* is the faradaic efficiency as percentage, and *Ecell* is the cell potential in V.

Energy consumption calculation:

$$\frac{kW*h}{ton} = \frac{E_{cell}*n*F}{MW*FE_{CO}*3.6}$$ Supplementary Equation 2

where *n* is the number of electrons transferred (dimensionless), *F* is Faraday´s constant in C mol^{-1}, *MW* is the molecular weight of CO in g mol^{-1}.

6. Comparison with literature

Supplementary Table 1. Experimental details of the different work from literature, used for the comparison made in Figure 5 in the main text. Only GDEs with geometrical surface area equal or larger than 5 cm² were considered.

	Cathode (GDE)	Anode	Electrolyte	CO_2 flow (mL min^{-1})	Load (mg cm^{-2})	Cathode area (cm²)	Ref
this work	Au nanoparticles	DSA®	1 M Cs_2SO_4	50	1	10	this work
Haas 2018	Ag GDE Covestro	Ir-MMO/Ti sheet	0.1 M K_2SO_4 + 1.5 M $KHCO_3$	52 and 10.5	n.a.	10	1
Verma 2018	MWNT/PyPBI/Au on Sigracet® 35 BC	IrO_2	2 M $KHCO_3$ or 2 M KOH	17	1	5	2
Verma 2016	Ag NP on Sigracet® 35 BC	IrO_2	1 M $KHCO_3$	17	2	10	3
Duarte 2019	Ag NP on Sigracet® 39 BC	platinized Ti	2 M $KHCO_3$	77	0.75	10	4
Jeanty 2018	Ag GDE Covestro	n.a.	0.4 M K_2SO_4	21	n.a.	7.67	5

Supplementary References

1. Haas, T., Krause, R., Weber, R., Demler, M. & Schmid, G. Technical photosynthesis involving CO_2 electrolysis and fermentation. *Nat. Catal.* **1**, 32–39 (2018).
2. Verma, S. *et al.* Insights into the Low Overpotential Electroreduction of CO_2 to CO on a Supported Gold Catalyst in an Alkaline Flow Electrolyzer. *ACS Energy Lett.* **3**, 193–198 (2018).
3. Verma, S., Lu, X., Ma, S., Masel, R. I. & Kenis, P. J. A. The effect of electrolyte composition on the electroreduction of CO_2 to CO on Ag based gas diffusion electrodes. *Phys. Chem. Chem. Phys.* **18**, 7075–7084 (2016).
4. Duarte, M., De Mot, B., Hereijgers, J. & Breugelmans, T. Electrochemical Reduction of CO_2: Effect of Convective CO_2 Supply in Gas Diffusion Electrodes. *ChemElectroChem* **6**, 5596–5602 (2019).
5. Jeanty, P. *et al.* Upscaling and continuous operation of electrochemical CO_2 to CO conversion in aqueous solutions on silver gas diffusion electrodes. *J. CO2 Util.* **24**, 454–462 (2018).
